# Comparative analysis of stress levels among working and non-working Indian women in rural Gujarat

**DOI:** 10.6026/973206300200735

**Published:** 2024-07-31

**Authors:** Mahalakshmi B., Sivasubramanian N., Sachin, Vihol Ujjavalben Rajendrasinh, Bhavariya Sushila Arunlal, Sathvara Rohit Dineshbhai, Chaudhari Sangitaben Meghabhai

**Affiliations:** 1 Nootan College of Nursing, Sankalchand Patel University, Visnagar, Gujarat - 384315, India

**Keywords:** Stress, women, employment status, rural areas, intervention, socio-demographic factors

## Abstract

Stress is derived from the Latin word "stringers" manifests as the body's response to various demands and pressures, affecting individuals' health and well-being. Therefore, it is of interest to evaluate stress levels in employed and unemployed women, recognizing the differential stress experiences in various life domains. A quantitative non-experimental comparative research design was employed, with data collected through structured questionnaires from 120 women in Visnagar, Gujarat. Results: Non-working women demonstrated lower stress levels compared to working women in pre-test measures. Post-intervention, non-working women experienced a reduction in stress, while working women showed no change. Demographic factors like age, education, and family structure did not significantly influence stress levels, except for monthly income, which correlated with lower stress across both groups. The study underscores significant disparities in stress levels between employed and unemployed women in rural Visnagar. Tailored interventions effectively reduced stress among non-working women but showed limited efficacy for working women. Financial stability emerged as a crucial factor in mitigating stress. Younger working women reported higher stress levels, suggesting the need for targeted interventions addressing career and familial pressures.

## Background:

The word "stress" is derived from a Latin word "stringers" that means, "to bind tight" and it is the shortened form of distress, which denotes noxious human experience. Stress, a ubiquitous phenomenon in modern society, manifests as the body's response to various demands and pressures, whether physical, emotional, or psychological [1]. While stress can serve as a natural adaptive mechanism, helping individuals cope with challenges, prolonged or excessive stress can have profound detrimental effects on health, well-being, and quality of life. Research suggests that women tend to experience higher levels of stress compared to men, often attributed to a multitude of factors such as societal expectations, gender roles, and the intersectionality of identity [2]. As per American psychiatric association October 2023 Stress in America survey, which included a nationally representative sample of more than 3,000 adults, women reported a higher average level of stress than men [5.3 versus 4.8 out of 10] and were more likely to rate their stress levels between an 8 and a 10 than men [27% versus 21%].men and women tend to react differently with stress-both psychologically and biologically. Working and non-working women can experience stress, but the sources of stress may differ. For working women, stress can stem from the demands of their jobs, managing work-life balance, workplace dynamics, career advancement pressures, and possibly juggling family responsibilities [3]. On the other hand, non-working women may experience stress related to managing household tasks, caring for children or other family members, financial concerns, societal expectations, or feelings of isolation or lack of fulfillment [4]. In today's fast-paced world, stress is everywhere. It affects everyone, but it hits women particularly hard. They often have to manage a lot of different roles, both at home and at work. Over the years, things have changed a lot for women, especially when it comes to work. While more opportunities have opened up for women in terms of jobs and education, it's also brought new challenges and pressures. For women with jobs, there's the pressure to do well at work while also dealing with office politics. For those who stay at home, there's stress too, like taking care of the family and meeting society's expectations. Therefore, it is of interest to evaluate the stress levels in both employed and unemployed women, acknowledging that stress presents itself differently in different areas of life.

## Methodology:

## Research design:

Our study employs a quantitative research approach.[5], utilizing a non-experimental comparative research design. This design was chosen to compare stress levels between working and non-working women without manipulating variables.

## Setting:

The research was conducted in selected areas of Visnagar, including Kansa, Kamana, and Savala villages.

## Participants:

Participants were recruited through convenience sampling, with individuals residing in the specified areas being approached for participation. Inclusion criteria included willingness to participate, availability during data collection, and proficiency in reading and writing Gujarati and English. The intended sample size was 120 participants, equally divided between working and non-working women.

## Instruments:

Data collection involved the use of a structured questionnaire consisting of demographic information and a stress questionnaire scale. The stress questionnaire assessed stress levels based on various factors, including general stress, causes, symptoms, and prevention.

## Data analysis:

Descriptive and inferential statistical analyses were conducted to analyze the collected data. Mean, standard deviation, and correlation analyses were used to examine relationships and patterns in the data. Statistical software SPSS 23 was utilized for data analysis. [6]

## Results:

Above graph shows that non-working women indicated 41 cases of stress, which decreased to 51 post-interventions. In contrast, working women showed no change in stress cases, maintaining a pre-test and post-test score of 0 for no stress. Mild stress increased slightly among working women from 6 to 10 cases, while non-working women saw a decrease from 14 to 4 cases. Moderate stress increased among both groups, with working women rising from 20 to 30 cases and non-working women remaining at 5 cases. Lastly, severe stress was solely reported by working women, with cases decreasing from 34 to 20 post-intervention. The mean score of stress among working women was 30.36 with a standard deviation of 9.74, while for non-working women, the mean score was 14.66 with a standard deviation of 5.24. The mean difference between the two groups was 15.7, and the correlation coefficient between working status and stress level was found to be r=0.5. Therefore, it was concluded that stress levels among working women were significantly higher compared to non-working women. The chi-square analysis was conducted to examine the relationship between stress levels and various demographic factors among both working and non-working women. Results revealed no significant associations between stress levels and age, education status, occupational status of the participant or their husband, working hours, religion, or type of family for both groups. This suggests that factors such as age, educational background, occupation, and family structure may not significantly influence stress levels in either working or non-working women. However, a notable finding was the significant association between monthly income and stress levels for both groups, indicating that higher monthly income is associated with lower stress levels. This underscores the potential importance of financial stability in reducing stress among women. Overall, while certain demographic factors may play a role in stress levels, income appears to be a more influential determinant across both groups.

## Discussion:

Our study reveals significant disparities in stress levels between working and non-working women in rural areas at Visnagar. Non-working women experienced a notable reduction in stress post-intervention, while working women showed no change, indicating the differential effectiveness of interventions. In the pretest results, our study indicated that non-working women experienced lower levels of stress compared to working women. This finding is consistent with the results of multiple studies conducted in similar contexts. For instance, a study by lee *et al.* (2023) found that employed women reported significantly higher stress levels than their non-working counterparts. [7] Thabassum *et al.* (2022) observed a similar trend, with non-working women demonstrating better stress management abilities and overall lower perceived stress levels compared to working women. [8] These findings collectively suggest that employment status may serve as a trigger factor for stress among women in various settings. The correlation coefficient between working status and stress level [r=0.5] further supports this association, highlighting the significant impact of employment status on stress levels among rural women. In our study, the pretest-post-test analysis revealed a notable reduction in overall stress levels among non-working women post-intervention, with the total number of stress cases decreasing from 41 to 51. This suggests that the intervention implemented effectively alleviated stress among non-working women in rural areas. This finding aligns with a study conducted by Sharma *et al.* (2023), which reported similar results of decreased stress levels among unemployed women following a targeted intervention program. [9] Conversely, working women in our study showed no change in stress cases post-intervention, maintaining a pretest and post-test score of 0 for no stress. However, there was a slight increase in mild stress cases among working women, from 6 to 10, indicating potential areas for improvement in addressing mild stressors in this group. This finding contrasts with the results of a study conducted by Kamaldeep *et al.* (2016), which reported a significant decrease in overall stress levels among employed women post-intervention. [10] The discrepancy in findings suggests that the effectiveness of interventions may vary depending on the specific characteristics and needs of the target population.

In terms of demographic factors, our chi-square analysis revealed no significant associations between stress levels and various demographic variables for both working and non-working women, except for monthly income. This finding suggests that factors such as age, education status, occupation, and family structure may not significantly influence stress levels in either group. However, the significant association between monthly income and stress levels for both groups underscores the importance of financial stability in mitigating stress among women in rural areas. This aligns with the findings of previous studies, which also reported a significant correlation between higher income levels an0d lower stress levels among women. [11] Our findings indicate that younger working women reported higher levels of stress, possibly due to the pressures of establishing their careers and balancing familial responsibilities. These findings align with the research conducted by Chengyue *et al.* (2023), which also found a significant association between age and stress levels among working women in rural settings. [12] Strengths of our study include its comparative design, allowing for the examination of stress levels across employment statuses and the inclusion of standardized instruments to assess stress levels. However, limitations include the reliance on self-reported data, potential confounding variables not accounted for, and the limited generalizability of findings beyond rural areas at Visnagar. In conclusion, our study underscores the need for tailored interventions to address differential stress experiences among working and non-working women in rural areas. These findings highlight the importance of considering employment status and socio-demographic factors in mental health interventions targeting women's well-being.

## Figures and Tables

**Figure 1:**
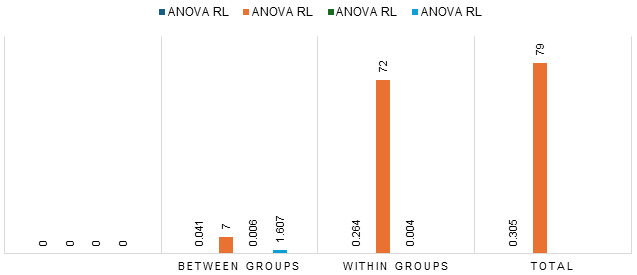
Comparison of pre-test & post-test stress level among working as well and non-working women

**Table 1:** Frequency Percentage and Distribution of the Selected Demographic Variables of the working women and non-working women, N = 120

**SR. NO.**	**DEMOGRAPHIC VARIABLES**	**WORKING WOMEN Frequency (%)**	**NON-WORKING WOMEN Frequency(%)**
1	Age		
	21-30	35(58.33%)	24(40%)
	31-40	19(31.66%)	22(36.66%)
	41-50	6(10%)	14(23.33%)
	50 Above	0(0%)	0(0%)
2	*Educational status*		
	Graduation	23(38.33%)	25(41.66%)
	Post-graduation	28(46.66%)	24(40%)
	Others	9(15%)	11(18.33%)
3	*Occupation of participant*		
	Government	25(41%)	21(35%)
	Semi-government	29(48.33%)	20(33.33%)
	Private	14(23.33%)	19(31.66%)
	Others	0(0%)	0(0%)
4	*Occupational status of husband*		
	*Government*	25(41%)	21(35%)
	Semi-government	29(48.33%)	20(33.33%)
	Private	14(23.33%)	19(31.66%)
	Others	0(0%)	0(0%)
5	*Working Hours*		
	06-Aug	35(58.33%)	33(55%)
	09-Nov	25(41.66%)	27(45%)
6	*Monthly income in rupees*		
	<5000	12(20%)	9(15%)
	6000-10000	18(30%)	20(33.33%)
	10000-15000	14(23.33%)	15(25%)
	>15000	16(26.66%)	16(26.66%)
7	*Religion*		
	Hindu	35(58.33%)	33(55%)
	Muslim	25(41.66%)	27(45%)
	Other	0(0%)	0(0%)
8	*Length of marriage life*		
	1-3 yr	35(58.33%)	33(55%)
	4-6 yr	25(41.66%)	27(45%)
	Above 7 yr	0(0%)	0(0%)
9	*Type of family*		
	Joint	35(58.33%)	33(55%)
	Nuclear	25(41.66%)	27(45%)
10	*Number of Children*		
	1	23(38.33%)	25(41.66%)
	2	28(46.66%)	24(40%)
	3 and above	9(15%)	11(18.33%)
